# Dual Microphone Voice Activity Detection Based on Reliable Spatial Cues

**DOI:** 10.3390/s19143056

**Published:** 2019-07-11

**Authors:** Soojoong Hwang, Yu Gwang Jin, Jong Won Shin

**Affiliations:** 1 School of Electrical Engineering and Computer Science, Gwangju Institute of Science and Technology, 123 Cheomdan-gwagiro, Buk-gu, Gwangju 61005, Korea; 2 AI Technology Unit, SK Telecom, 100 Eulji-ro, Jung-gu, Seoul 04551, Korea

**Keywords:** dual microphone, interchannel level difference, interchannel time difference, frequency selection, voice activity detection

## Abstract

Two main spatial cues that can be exploited for dual microphone voice activity detection (VAD) are the interchannel time difference (ITD) and the interchannel level difference (ILD). While both ITD and ILD provide information on the location of audio sources, they may be impaired in different manners by background noises and reverberation and therefore can have complementary information. Conventional approaches utilize the statistics from all frequencies with fixed weight, although the information from some time–frequency bins may degrade the performance of VAD. In this letter, we propose a dual microphone VAD scheme based on the spatial cues in reliable frequency bins only, considering the sparsity of the speech signal in the time–frequency domain. The reliability of each time–frequency bin is determined by three conditions on signal energy, ILD, and ITD. ITD-based and ILD-based VADs and statistics are evaluated using the information from selected frequency bins and then combined to produce the final VAD results. Experimental results show that the proposed frequency selective approach enhances the performances of VAD in realistic environments.

## 1. Introduction

Voice activity detection (VAD) which decides if the speech signal is present in the current frame of the input signal has become a crucial part of the speech enhancement, noise estimation, pitch extraction, and the variable rate speech codecs [1,2,3,4,5,6,7,8,9,10,11,12,13,14,15,16,17,18,19,20,21,22,23,24,25,26,27,28,29,30,31,32]. Single microphone VAD usually utilizes energy-related features, statistical model-based statistics including signal-to-noise ratios (SNRs) and likelihood ratios [8,9,10,11], or speech-specific features such as linear predictive coefficients, formant shape, zero-crossing rate, and cepstral features [12,13,14,15,16,17,18,19,20]. Recently, single microphone VADs using deep neural networks are also proposed [22,23,24,25,26], although some of the application scenarios such as mobile devices may not accommodate much computation. These single channel approaches, however, cannot utilize spatial diversity of the sound sources and therefore the performance is limited when strong speech-like interference is present. Since the devices with multiple microphones have become popular these days, reliable multi-microphone VAD becomes more important.

Like the human binaural perception, two main cues that capture the spatial information may be the differences of the arrival times and signal levels in the microphones. These statistics, which are called interchannel time difference (ITD) and interchannel level difference (ILD), can be exploited for dual microphone VAD. When the range of the direction-of-arrival (DoA) of the desired signal is known in advance as in the case of the handset mode of the mobile phone, the VAD can be constructed based on the estimated DoAs. There have been several approaches to the DoA estimation and the VAD based on ITD. The generalized cross correlation with phase transform (GCC-PHAT) [33] may be the most popular approach for DoA estimation and it can be utilized for ITD-based VAD. The long term information of interchannel phase difference (LTIPD) was also proposed as a test statistics for ITD-based VAD, which measures how consistently the signal energy is concentrated in a small DoA range [27]. ILD can also be utilized for VAD when the target source is located close to one of the microphones or there are obstacles between the target source and one of the microphones. In [28], the normalized difference of power spectral density (NDPSD) was proposed as a test statistic for VAD. Choi and Chang [29] proposed two step power level difference ratio (PLDR) using two different smoothing factors as an alternative measure of ILD. There have also been approaches exploiting both ITD and ILD information, as they reflect different characteristics of spatial diversity. In [30], voice activity is decided using a support vector machine (SVM) for which the inputs include both ITD-based and ILD-based features. This approach can configure the relative importance of the ILD or ITD related features in different frequency bins based on the training data, but cannot modify it dynamically according to the input signal. Statistical model-based approaches were also proposed for multichannel VADs adopting complex Gaussian model [31] or the spherically invariant random process [32] for the distribution of each frequency component.

As speech signal is sparse in the time–frequency (TF) domain in nature, there are always TF bins with SNRs in which the ILD or ITD information are more reliable and those with low SNRs where the ILD or ITD information may not be useful at all in noisy environments. In this letter, we propose a VAD based on both ITD and ILD information from reliable frequency bins only. The reliability is determined for each TF bin by three conditions on signal energy, ILD, and ITD. ITD-based and ILD-based VADs or statistics evaluated using only the reliable frequency bins are combined together to construct final voice activity decision. The long term spectral divergence [17] and the subband order statistics filter [18] uses the energies in the specific percentiles among the neighboring frames for the given subband to evaluate the statistics for VAD. In [21], a subset of the frequency bins are utilized for the SNR-based VAD, but the selection criterion is only based on the energies unlike the proposed approach. Experimental results showed that the proposed approach can enhance the performance of the dual microphone VAD.

## 2. Dual Microphone VADs Based on Spatial Cues

The dual microphone VAD can perform better if prior knowledge on the location of the desired speaker is available. One example of the scenarios in which the range of the locations of the desired signal source is known in advance is the handset mode of the mobile phone, where user’s mouth is much closer to the primary microphone.

Let $Y_{1}\left(l,k\right)$ and $Y_{2}\left(l,k\right)$ be the *K*-point short-time Fourier transform (STFT) coefficients of the signals from the primary and secondary microphones for the *k*th frequency bin at the *l*th frame, respectively. The test statistics for the NDPSD-based VAD [28] are the difference of the powers normalized by the average power of two microphone signals, which are not dependent on the absolute signal level. The NDPSD for each frequency bin is given as

(1) $$\Delta \Phi (l,k)=\frac{|Y_{1}{\left(l,k\right)|}^{2}-{\left|Y_{2}\left(l,k\right)\right|}^{2}}{|Y_{1}{\left(l,k\right)|}^{2}+{\left|Y_{2}\left(l,k\right)\right|}^{2}},$$

where |·| denotes the magnitude. The voice activity is decided by comparing the NDPSD averaged over all frequency bins with a threshold $\xi_{NDPSD}$ as

(2) $$\begin{array}{cc} V_{NDPSD}\left(l\right) & =\left\{\begin{array}{cc} 1, & \mathrm{if}\frac{2}{K}\sum_{k=1}^{K/2}\Delta \Phi (l,k)\geq \xi_{NDPSD}\\ 0, & \mathrm{otherwise} \end{array}\right.. \end{array}$$

It is noted that each frequency bin contributes to the final test statistic equally, although the absolute values of ΔΦ(l,k) do not have much information on the presence of speech in the current frame if ΔΦ(l,k) is negative.

The two step PLDR method [29] also takes the difference of the powers in microphone signals as basic information. The power differences of the input signals and noises in the microphones for each frequency are recursively smoothed with two different smoothing factors to produce long-term and short-term smoothed input and noise power level difference, ${\hat{\Delta P}}_{Y}^{LT}\left(l,k\right)$, ${\hat{\Delta P}}_{Y}^{ST}\left(l,k\right)$, ${\hat{\Delta P}}_{N}^{LT}\left(l,k\right)$, and ${\hat{\Delta P}}_{N}^{ST}\left(l,k\right)$, with the help of estimated speech presence probability. Then, the $log\frac{{\hat{\Delta P}}_{Y}^{j}\left(l,k\right)}{{\hat{\Delta P}}_{N}^{j}\left(l,k\right)}$ are averaged over all frequency bins to produce two PLDRs. They are converted to two *a posteriori* probabilities of speech presence, and then thresholded to determine voice activity. Like NDPSD, PLDRs can also be strongly affected by the presence of the interfering source near the secondary microphone.

Another important source of information about the location of the sound sources is ITD. Ref. [27] proposed LTIPD which measures how much energy is concentrated in the frequency bins for which the DoA estimates for nearby frames fall into the same small DoA sector. The target DoA range is divided into *U* overlapped sectors of which the width are equal. LTIPD is then defined as

(3) $$E\left(l\right)=\max_{1\leq i\leq U}\sum_{k:C_{i}\left(l,k\right)>\kappa_{i}}{\left|Y_{1}\left(l,k\right)\right|}^{2},$$

where *i* denotes the indices of the sectors, $C_{i}\left(l,k\right)$ is the number of frames in which DoA estimate for the *k*-th frequency bin indicates the *i*th sector among the last *L* frames, and $\kappa_{i}$ is the threshold of the concentration of DoAs. The VAD based on LTIPD is given as

(4) $$\begin{array}{cc} V_{LTIPD}\left(l\right) & =\left\{\begin{array}{cc} 1, & \mathrm{if}\;E\left(l\right)\geq \xi_{LTIPD}\\ 0, & \mathrm{otherwise} \end{array}\right.. \end{array}$$

It is noted that the LTIPD-based VAD includes the reliable frequency selection, but the selection criterion is based only on the ITD information.

The performance of VAD may be improved by utilizing both ITD and ILD information simultaneously, as two statistics provide different information on the spatial diversity. In [30], the VADs based on both ITD and ILD information are proposed. The simplest way proposed in [30] is the logical combination of the ITD-based and ILD-based VADs. The VAD using the logical “and” operation of the voice activities from ITD and ILD is given as

(5) $$\begin{array}{cc} V_{AND}\left(l\right) & =\left\{\begin{array}{cc} 1, & \mathrm{if}\;V_{ITD}\left(l\right)=1\;\mathrm{and}\;V_{ILD}\left(l\right)=1\\ 0, & \mathrm{otherwise} \end{array}\right., \end{array}$$

which was found to be more effective than the “OR” operation from several experiments. Among the candidates for the $V_{ITD}$ and $V_{ILD}$, the combination of the LTIPD-based VAD and the NDPSD-based VAD performed the best [30]. Another method proposed in [30] is to build a VAD using SVM for which the input includes both the ILD-based and ITD-based features. After training with the clean speech data mixed with various noises, the output of the SVM, $y\left(x(l)\right)=w_{o}^{T}x\left(l\right)+b_{o}$ where x(l) is the input of the SVM and $w_{o}$ and $b_{0}$ are the weight vector and the bias for the optimal hyperplane given the training set, respectively [34], is used to estimate the a posteriori probability $p(V_{SVM}=1|x\left(l\right))$ in the test phase. Then, the decision rule becomes

(6) $$\begin{array}{cc} V_{SVM}\left(l\right) & =\left\{\begin{array}{cc} 1, & \mathrm{if}\;p\left(V_{SVM}=1|x\left(l\right)\right)\geq \xi_{SVM}\\ 0, & \mathrm{otherwise} \end{array}\right.. \end{array}$$

As for the input of the SVM, it is reported that $|Y_{1}{\left(l,k\right)|}^{2}$, $|Y_{2}{\left(l,k\right)|}^{2}$, and the phase difference between $Y_{1}\left(l,k\right)$ and $Y_{1}\left(l,k\right)$, Δψ(l,k), showed the best performance among a number of ILD- and ITD-related features [30]. However, in our experiments, including $p_{LT}\left(l\right)$ and $p_{ST}\left(l\right)$ from the two-step PLDR approach on top of $|Y_{1}{\left(l,k\right)|}^{2}$, $|Y_{2}{\left(l,k\right)|}^{2}$, and Δψ(l,k) slightly improved the performance of $V_{SVM}$. Although the SVM can put different importance on the statistics from different frequency bins, the weights are fixed in the test phase and cannot be dynamically changed from frame to frame.

The performance of each VAD can be significantly enhanced by introducing the hangover scheme, which requires several consecutive frames with instantaneous VAD of 0 to make the final VAD to be 0. The number of hangover frames remains as a tunable parameter along with the thresholds for each VAD. For $V_{AND}$, the hangover scheme is applied when evaluating $V_{ITD}$, $V_{ILD}$, and $V_{AND}$ with three separate hangover parameters.

## 3. Dual Microphone VAD Using Reliable Spatial Cues

As speech components are sparsely distributed in the TF domain, the spatial cues in some of the TF bins are useful while those from other TF bins are not reliable. In this paper, we propose a dual microphone VAD based on the spatial information from the selected frequency bins with high reliability, which is determined in each frame by signal energies, ILDs, and ITDs for the corresponding frequency bins. After we determine the reliability of information from each TF bin, the test statistics for conventional VAD approaches are modified to consider reliable TF bins only.

Let us denote the spectral mask to select reliable frequency bins for the *k*-th frequency bin at the *l*-th frame as m(l,k), i.e., m(l,k)=1 for the TF bins with reliable spatial information and m(l,k)=0 for other TF bins. The first condition to determine the reliability of the spatial information in each TF bin is on the signal energy. If the energy of the input noisy signal in the primary microphone is not high enough, the probability of speech presence is low and the ILD and ITD information is vulnerable to measurement noises. Thus, the first sub-mask $m_{1}\left(l,k\right)$ is constructed based on the input signal energy as follows:

(7) $$\begin{array}{cc} m_{1}\left(l,k\right) & =\left\{\begin{array}{cc} 1,\; & \mathrm{if}\;|Y_{1}{\left(l,k\right)|}^{2}\geq \eta_{1}\\ 0, & \mathrm{otherwise} \end{array}\right., \end{array}$$

where $\eta_{1}$ is the threshold.

The second and third conditions determine the reliability in each bin with ILD and ITD information, respectively. The second sub-mask $m_{2}\left(l,k\right)$ becomes 1 if the instantaneous ILD for the frequency is high enough:

(8) $$\begin{array}{cc} m_{2}\left(l,k\right) & =\left\{\begin{array}{cc} 1,\; & \mathrm{if}\;\log\frac{|Y_{1}{\left(l,k\right)|}^{2}}{|Y_{2}{\left(l,k\right)|}^{2}}\geq \eta_{2}\\ 0, & \mathrm{otherwise} \end{array}\right., \end{array}$$

where $\eta_{2}$ is the threshold for the level difference. Since this sub-mask is only used to select reliable spectral bins from which the test statistics for the dual microphone VAD are computed, the threshold $\eta_{2}$ is not set to maximize the performance of the VAD, but is configured to discriminate the frequency bins that may contain desired speech signals and those in which there is definitely no speech signal. This sub-mask would enhance the robustness of the VAD to the noise sources that are close to the secondary microphone, which may have a huge adverse impact on the ILD statistics. The frequency bins with low enough ILD will not have the desired speech and should be excluded in the computation of the ITD-based test statistics. The third condition based on ITD also aims to eliminate the TF bins in which the speech is absent for sure. The third sub-mask $m_{3}\left(l,k\right)$ is 1 only if ITD in the TF bin is between $\tau_{1}$ and $\tau_{2}$ which correspond to the time differences of arrival (TDoAs) when the source is located at the boundaries of the target DoA range:

(9) $$\begin{array}{cc} m_{3}\left(l,k\right) & =\left\{\begin{array}{cc} 1, & \mathrm{if}\;\tau_{1}\leq \frac{\Delta \psi (l,k)K}{2\pi f_{s}k}\leq \tau_{2}\;\mathrm{or}\;k>K_{2}\;\mathrm{or}\;k<K_{1}\\ 0, & \mathrm{otherwise} \end{array}\right., \end{array}$$

where ($K_{1}$, $K_{2}$) is the range of the frequency bin index. $K_{1}$ is set to exclude low frequency bins in which the DoA estimate is too sensitive to the small errors in phase measurement, whereas $K_{2}$ is set to avoid spatial aliasing with a certain margin. It essentially means that we will not consider ILD or ITD information from the TF bins for which the TDoA is out of $\left(\tau_{1},\tau_{2}\right)$ range. The combined spectral mask is given as $m\left(l,k\right)=m_{1}\left(l,k\right)m_{2}\left(l,k\right)m_{3}\left(l,k\right)$, which passes through only the spectral information from TF bins satisfying all the three conditions to prevent the disturbance from the TF bins with interferences only. In addition, the frames with few valid bins are regarded as non-speech frames, i.e., the final voice activity is 0 if the number of valid frequency bins, $K_{m}\left(l\right)=\sum_{k}m\left(l,k\right)$, is less than a certain threshold, $K_{min}$. It is noted that $\eta_{1}$, $\eta_{2}$, $\tau_{1}$, and $\tau_{2}$ are set to reject only TF bins with definitely no speech signals considering the following combination with other masks.

The proposed frequency selective approach using the spectral masks m(l,k) can be applied to any frequency domain VAD methods that summarize the information from each frequency. The frequency selective version of the NDPSD-based VAD becomes

(10) $$\begin{array}{cc} V_{NDPSD}^{FS}\left(l\right) & =\left\{\begin{array}{cc} 1, & \mathrm{if}\;\sum_{k=1}^{K/2}\frac{m(l,k)}{K_{m}\left(l\right)}\Delta \Phi (l,k)\geq \xi_{NDPSD}\;\mathrm{and}\;K_{m}\left(l\right)\geq K_{min}\\ 0, & \mathrm{otherwise} \end{array}\right.. \end{array}$$

Similarly, the Equation (3) for the LTIPD-based VAD is modified to consider only reliable frequency bins as $E^{FS}\left(l\right)=\max_{1\leq i\leq U}\sum_{k:C_{i}\left(l,k\right)>\kappa_{i}}m\left(l,k\right){\left|Y_{1}\left(l,k\right)\right|}^{2}$, and then $V_{LTIPD}^{FS}\left(l\right)$ can be constructed in a similar manner with the Equation (4). It is not straightforward to modify the two step PLDR method incorporating m(l,k). The adaptation of short-term and long-term smoothing factors are originally governed by the speech presence probability, which is computed based on the ILD and the ILD for noise only period. As the smoothing factor control part can be viewed as a soft-decision version of the frequency selective approach although it relies solely on ILD information, we did not construct the frequency selective version of it. The frequency selective version of $V_{AND}$ is simply obtained by “AND” operation of $V_{NDPSD}^{FS}\left(l\right)$ and $V_{LTIPD}^{FS}\left(l\right)$. As for the VAD based on SVM, the input features corresponding to the TF bins with m(l,k)=0 are set to 0 so that they do not contribute to the output of the SVM, $y\left(x\left(l\right)\right)=w_{o}^{T}x\left(l\right)+b_{o}$. Then, $V_{SVM}^{FS}\left(l\right)$ is constructed based on the *a posteriori* probability computed from the output of the SVM with masked input vectors.

## 4. Experimental Result

**Figure 1:**
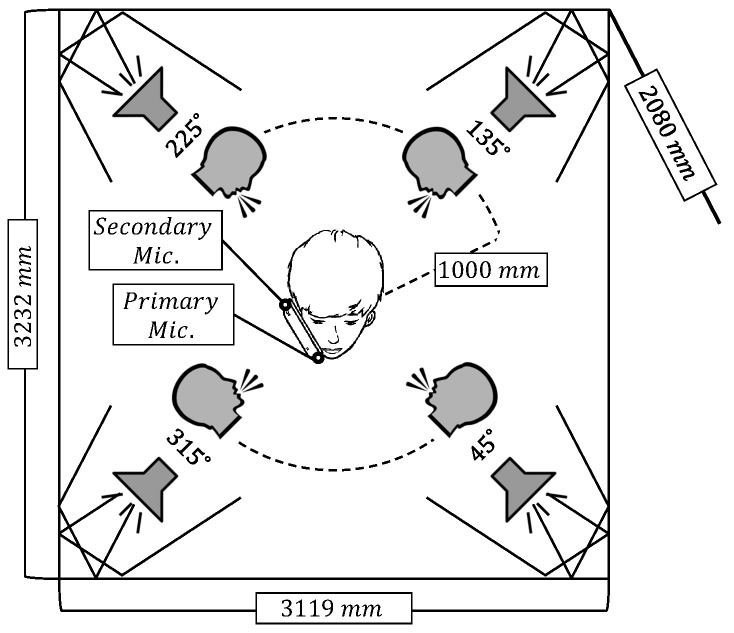
Geographical placement of the noise sources and the receiver.

To demonstrate the performance of the proposed algorithm, we have recorded the desired speech, directional interferences, and diffuse noises with two microphones located on a commercial mobile phone, Samsung Galaxy S7. The placement of the user and the loudspeakers are illustrated in Figure 1. The size of the room was 3119 × 3232 × 2080 mm^3^ and the reverberation time of the room was approximately 120 ms. In the center of the room, one of the two male and two female speakers stood holding the mobile phone with the right hand in the handset mode. The desired near-end speech was spoken by those speakers in English. The distance between the microphones was about 140 mm. The diffuse noise field was generated by playing back white, babble, or car noises from NOISEX-92 database [35] with four loudspeakers facing the corners of the room to incur complex reflections as shown in Figure 1. Directional interferences were the speech utterances spoken by four male and four female speakers chosen from the TIMIT database, and were played from one of the four loudspeakers located 1000 mm away from the user at the directions {45°, 135°, 225°, 315°} facing the users as depicted in Figure 1. Two minutes of near-end speech spoken by one male and one female speakers was used for the training of the SVM and the parameter setting of other approaches, while another six minute long speech spoken by the other male and female speakers was used to generate the test data. The near-end speech was mixed with a directional interference coming from one of the four loudspeakers or one of the diffuse noises at the SNR level out of {−5, 0, …, 20} dB on the primary microphone, which makes the total length of the training and test data 84 and 252 min, respectively. The sampling rate was 8 kHz, and 256 point Hamming window was applied with 10 ms frame shift. The frequency index range ($K_{1}$, $K_{2}$) considered in $m_{3}\left(l,k\right)$ and LTIPD was (4, 31), which corresponds to (125 Hz, 968.75 Hz), considering that the microphone distance was 140 mm. The range of the target DoA for the ITD-based VAD statistics was set to be (10°, 70°) when 0° corresponds to the end-fire direction to the primary microphone side. The range of the target DoA for the reliable frequency selection that determines $\tau_{1}$ and $\tau_{2}$ in Equation (9) was (0°, 80°), which was a bit wider than that for VAD. These values were set to allow the deviation of the estimated DoA due to the noises and the individual variability in how to hold the phone. The thresholds for $m_{1}\left(l,k\right)$ and $m_{2}\left(l,k\right)$, $\eta_{1}$ and $\eta_{2}$, were set to minimize the equal error rate on training set. The parameters for the LTIPD-based VAD, *L* and *U*, were set to 12 and 10, respectively. $K_{min}$ was set to 3.

**Figure 2:**
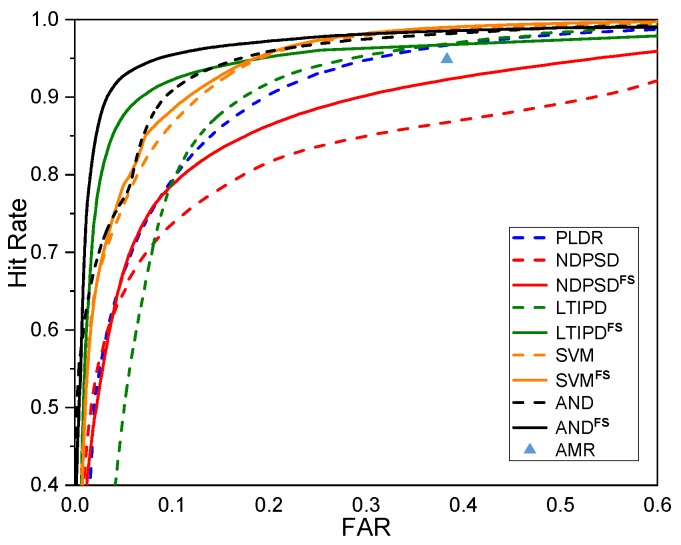
Receiver operating characteristics curves for various voice activity detection (VAD) with and without the proposed frequency selective approach.

**Figure 3:**
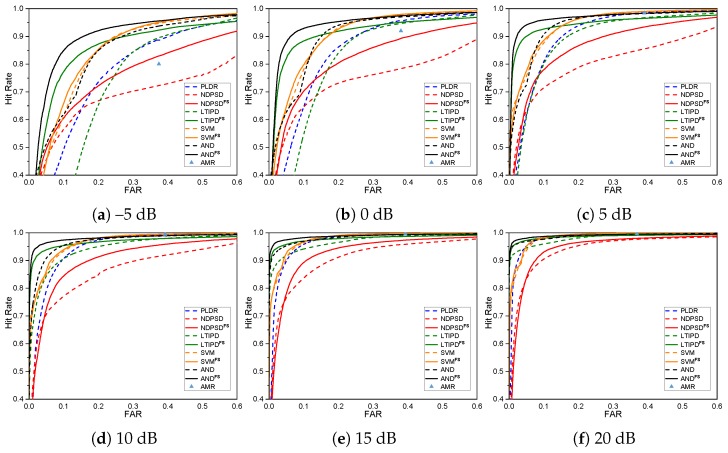
Receiver operating characteristics curves for various VADs with and without the proposed frequency selective approach for each signal-to-noise ratio (SNR).

The performances of the various ILD- and ITD-based VAD methods with and without the proposed reliable frequency bin selection for the whole test data are shown in Figure 2 in the form of the receiver operating characteristics (ROC) curves. The original ROC curves are the collection of the (Hit rate, FAR) pairs with different threshold parameters, so one ROC curve can be drawn for each number of hangover frames for each VAD. The curves shown in Figure 2 are the collection of the leftmost points among all ROC curves with different hangover parameters given the hit rate for each VAD method. Adaptive multi-rate (AMR) VAD option 2 was also shown as a performance benchmark [7]. The performances of the original VAD methods using all frequency bins are shown as dashed lines, while the solid lines indicate the performances of the frequency selective versions of the corresponding methods. The VADs with the proposed frequency selective approach outperformed the original VAD methods. Among all the methods, $V_{AND}^{FS}$, the frequency selective version of $V_{AND}$, performed the best when the false alarm rate (FAR) was less than 29.02%, while $V_{SVM}^{FS}$ was the best when we need a hit rate higher than 98.06% in the expense of a higher FAR. Figure 3 shows the ROC curves for different SNRs averaged over all noise types. The tendency that the reliable frequency selection improves the performance was similar in all cases except some regions with very low hit rates. The performance difference between $V_{AND}$ and $V_{SVM}$ was not as large as in [30], possibly due to the mismatch in the near-end speakers in the training and the test. $V_{SVM}^{FS}$ could further enhance the performance of the $V_{SVM}$ with dynamic frequency selection, especially for the lower SNRs. Table 1 shows the performance of each VAD in terms of accuracy, precision, and recall at the operating point minimizing the overall error rate $E_{OVR}$ defined as $E_{OVR}=\alpha \times FRR+\left(1-\alpha \right)\times FAR$ [30], in which FRR denotes the false rejection rate. *α* was set to 0.8 as the false rejection may be more critical for variable rate speech codecs or speech enhancement. Experimental results in a more reverberant environment are also available at the demo page (https://mspl.gist.ac.kr/vad_demo/dualchannelVAD.html).

## 5. Conclusions

In this letter, we have proposed a dual microphone VAD scheme based on the spatial information from reliable TF bins only. The frequency selection masks are constructed based on three criteria on signal energy, ILD, and ITD. Conventional ITD- and ILD-based VADs and the combination of them are modified to consider the spatial information from the reliable spectral bins only. Experimental results with a commercial smartphone showed that the frequency selective approach can enhance the performance of the ILD- and ITD-based VADs, the logical combination of them, and the SVM-based VAD in various noise environments.

## Figures and Tables

**Table 1:** Performance of accuracy, precision, and recall at the operating point minimizing $E_{OVR}$.

METHOD	AMR	PLDR	NDPSD	NDPSD^FS^	LTIPD	LTIPD^FS^	SVM	SVM^FS^	AND	AND^FS^
**Accuracy**	83.28	94.35	87.79	90.19	93.38	96.17	94.40	94.46	96.42	97.13
**Precision**	76.91	92.09	84.14	87.48	92.37	95.83	92.16	92.11	96.93	97.00
**Recall**	99.64	98.20	95.97	95.97	95.94	97.31	98.22	98.40	96.58	97.82
